# Evaluation of the pharmacy practice program in the 6-year pharmaceutical education curriculum in Japan: community pharmacy practice program

**DOI:** 10.1186/s40780-015-0026-3

**Published:** 2015-09-30

**Authors:** Miho Utsumi, Sachi Hirano, Yuki Fujii, Hiroshi Yamamoto

**Affiliations:** Faculty of Pharmaceutical Sciences, Kobe Gakuin University, Chuo-ku, Kobe-shi 650-8586 Hyogo, Japan

**Keywords:** Pharmacy, Hospital, Curricula, Learning, Training

## Abstract

**Background:**

The purpose of this study was to highlight concerns with the current pharmacy practice program and suggest aspects for improvement. A further aim of the study was to enhance the educational effects of the program, from the students’ point of view.

**Methods:**

We surveyed 1,607 pharmacy students in Japan who had completed the pharmacy practice program in either 2010 or 2011. The students completed a self-descriptive questionnaire comprising 48 questions examining their experience of the pharmacy practice program.

**Results:**

For community pharmacy practice, four factors were extracted through exploratory analysis: “satisfactory learning (pharmacy),” “support system of the university,” “creation and clarification of the training plan,” and “dialogue with patients.” When comparing the mean values for each of the four factors between 2011 and 2012, the 2012 group scored significantly higher (*p* < 0.001) on “support system of the university” only. In the free responses, it became apparent that, for the joint training held in certain regions, students evaluated such training to be useful and effective. Moreover, we conducted an overall evaluation of the pharmacy practice programs. From the results of McNemar’s test, from 2011 to 2012, there was a significant decrease in the number of students who were unable to experience “charge system of patients” at neither hospitals nor pharmacies (*p* < 0.01).

**Conclusions:**

For community pharmacy practice, there were no significant differences found for the factors, with the exception of the “support system of the university.” In addition, to accomplish the learning objectives, community pharmacy practice program introduced some initiatives. Furthermore, conducting training at multiple facilities deepens student learning and assists with the correction of problems, such as the disparities within the teaching system and learning content at each of the training facilities.

## Background

The advancement of medical and scientific technology, and the separation of medical and dispensary practices, has brought about a tremendous change in the state of pharmaceuticals in Japan. In order for pharmacists to appropriately respond to such needs, students aspiring to become pharmacists must acquire basic knowledge/skills, an appreciation for humanity, respectable ethics, medical knowledge, practical skills, and have the ability to detect and solve problems [1].

To meet these new requirements, the curriculum was changed to a six-year program in 2006. All Japanese universities initiated the new education system, based on the model core curricula for the pharmacy education/pharmacy practice programs [2, 3]. There has also been an expansion in the liberal arts and clinical pharmacy subjects included in the program [4–7]. Furthermore, unique subjects relating to pharmaceutics in universities have also been included [8–11].

In addition, the focus of the teaching methods switched from “teacher-centered” to “learner-centered” [12, 13]. Furthermore, methods using standardized patients and simulators are frequently employed in training to ensure high-quality performance, integration of knowledge and skills, and a positive attitude among pharmacists on-site [14–16]. The abovementioned educational methods have become the norm and all facilities throughout Japan are now required to continue evaluating and improving pharmaceutical education.

Various assessments of pharmacy practice programs have been conducted in different regions and facilities since their commencement in 2010. For example, Tachi et al evaluated the guidance skills of pharmacy practice instructors, while Mohri et al evaluated the necessity of resident teachers in hospital pharmacy practice [17, 18]. Furthermore, Yamaguchi et al revealed that training content is strongly dependent on the passion and appeal of the instructor. They further found that such qualities also influence students’ improvement, in terms of future learning motivation and the career paths they choose [19]. In order to conduct homogenous training, Kubo et al created a ward training pass; a weekly report of training content, such as pharmacotherapy and diseases, that trainees should be concerned with in each hospital department, and reported on its effectiveness [20].

However, a nationwide survey designed to determine the actual state of current pharmacy practice programs has still not yet been conducted. Furthermore, pre-practice training is promoted at universities and sets its learning objectives with “basic knowledge, skills, and attitude required for pharmaceutical duties” with regard to the hospital pharmacy practice and community pharmacy practice learning objectives of “team medical care” and “community-based care,” respectively [3]. The goals that must be achieved by pharmaceutical students, based on the present and expected state of future Japan, such as competency, have not been previously clarified.

Due to the above, we assessed hospital pharmacy practices in the six-year pharmaceutical education program. It was found that the student evaluations for the quality of hospital pharmacy practice improved in 2012, as compared to the evaluations from 2011 [21]. Furthermore, it was found that, in order for students to obtain “satisfactory learning (hospital)” in pharmacy practice programs, it is necessary to enhance the “support system of the training site (hospital)” and establish opportunities for “dialogue with patients.” The results further suggest that “dialogue with patients” leads to feelings of satisfaction, being able rise to a challenge and a sense of accomplishment among students. However, it was also found that the instructing ability of pharmacists, readiness to receive trainees at training facilities, and relationships between pharmacists and students still require further improvement.

Accordingly, our study clarifies the current state of community pharmacy practice and outlines the improvements that are needed to enhance current pharmacy practice programs. In addition to the findings from our research into both community pharmacy practice and hospital pharmacy practice, we have evaluated the overall state of current pharmacy practice programs. Furthermore, based on real-life medical settings in Japan, we have examined the ideal state of pharmacy practice programs for the future.

## Methods

The survey was conducted from September, 2011 to March, 2012 (hereafter referred to as “2011”) and from September 2012 to March 2013 (hereafter referred to as “2012”), and included 1,607 pharmacy students (5^th^ or 6^th^ year students; approximately 7.9 % of the nationwide total of 20,389 students) in Japan who completed pharmacy practice programs (pharmacy and hospital pharmacy practice) in 2010 or 2011. The sampling of the target students was used to determine the actual state of pharmacy practice programs performed nationwide in Japan. The study sampled target schools and was devised in a way to ensure a collection of nationwide responses.

The questionnaire used was similar to that used for the evaluation of hospital pharmacy practice, with the terms in the questions regarding learning objectives, hospitals and pharmacies, and individual examples, being replaced or adjusted as necessary. In addition to the questions on basic attributes, the structure of the questionnaire comprised 48 questions, including 31 questions based on a six-point scale (1: Disagree Strongly, 2: Disagree Moderately, 3: Disagree Slightly, 4: Agree Slightly, 5: Agree Moderately, 6: Agree Strongly), 15 questions based on a two-point scale (yes or no), one multiple choice question, and one open question (free response). The questionnaire included items relating to the pharmacy practice content, based on the students’ actual experience, such as “The actual training contents were in line with the learning objectives” and “I was able to experience the overall work of a pharmacist sufficiently.” There were also items relating to the learning environment, such as “I felt that there were too many tasks that would not directly lead to my learning as a trainee” and “When screening of prescriptions or dispensing, two pharmacists were involved to ensure the safety.” Moreover, the items that seemed to contribute to deepening the learning were included; “Did you have a chance to have discussions/reflection sessions among students?” Furthermore, we made an inquiry regarding the occurrence of problems at the training site, by asking “Did you have any trouble with a patient?” and regarding the training status of the pharmacist instructors, by asking “The pharmacist (who was involved in your training) worked very hard in coaching you” and “The pharmacists were able to have an empathetic communication with you.” Another item, regarding the support status of the university teachers, was included by asking “The university teachers provided enough support for me so that I can prevent trouble, or work on the practical training smoothly in case of trouble.” For questions on the training environment that were provided by pharmacists, the target was not limited to the licensed instructor pharmacists for pharmacy practice programs. Rather, we requested that students answer the questions keeping all involved pharmacists in mind.

For any missing values among the six-point scale items, the average value of the overall applicable items was substituted, while missing values for questions based on the two-point scale were removed from the analysis. For the 31 items based on a six-point scale, we conducted an exploratory factor analysis (maximum likelihood estimation/promax rotation) to clarify the factor structure of the overall responses. Next, we divided the obtained factor scores into two groups (2011 and 2012) and compared both years with Levene’s test. Homogeneous dispersal of the obtained factor points was not found on some factors. Thus, we conducted a *t* test based on Welch’s method, in order to compare the two years. For the items based on a two-point scale, we conducted a comparative evaluation of the two years using simple tabulation and the chi-square test.

In order to gain an understanding of the overall perception of the five-month pharmacy practice programs in hospitals and pharmacies (two and a half months for each location), we categorized the student response patterns to the items based on a two-point scale, and then conducted McNemar’s test on the data. We used R × 64 3.1.0, js-STAR release 2.0.6j, and Excel 2007 for statistical analysis.

Our study was conducted with the approval of the Ethics Committee of Kobe Gakuin University. Additionally, we explained that there was absolutely no influence on academic records without participation in the survey.

## Results

### Respondent attributes

The respondents of the survey totaled 1,407 (the effective response rate was 87.6 %) and the average age was 24.1 ± 1.5 years old (in 2011: 24.2 ± 1.8 years, in 2012: 24.1 ± 1.4 years). The student characteristics are shown by year in Table 1. Respondents who omitted basic characteristics (age, sex) and those that left three or more items blank were removed from the analysis.

**Table 1:** Attributes of the respondents

		Respondents (%)	
		2011	2012
Sex			
	Male	212(42)	376(41)
	Female	287(58)	532(59)
Area			
	Hokkaido	―	―
	Tohoku	―	―
	Kanto	133(27)	568(62)
	Hokuriku	―	―
	Tokai	20(4)	0(0)
	Kinki	194(39)	89(10)
	Chugoku/Shikoku	31(6)	114(13)
	Kyusyu/Yamaguchi	121(24)	137(15)
		499(100)	908(100)

### Factor analysis

We conducted an exploratory factor analysis targeting 31 items. This was based on a six-point scale utilizing maximum likelihood estimation/promax rotation, using the combined data from 2011 and 2012. For the number of factors, based on the size of the fixed value and with potential analysis as a factor, four-factor solutions were judged as the ideal. Twelve items that were indicative of being influenced by the ceiling and floor effect were removed from the analysis. Factor analysis was conducted repeatedly until a stable factor was extracted based on a factor loading of 0.35 and above.

Therefore, 19 analysis target items and a total of four factors were extracted (Table 2). The correlation between the factors revealed particularly high scores between the first and third factor, and the first and fourth factor (0.49 and 0.50, respectively). In addition, we calculated Cronbach’s α coefficient between the items that formed each factor, to evaluate the internal consistency of the scale. The factors indicated comparatively high values and the reliability of each factor was confirmed: first factor α = 0.74, second factor α = 0.75, third factor α = 0.70, and fourth factor α = 0.72. To ensure that the data from the pilot study can be put to good use, from the viewpoint of evaluating interpretable information without discarding it as much as possible, we utilized a standard (factor loading > 0.35) which is slightly lower than the general standard (factor loading > 0.40).

**Table 2:** Results of the factor analysis for the community pharmacy practice

Question	FA1	FA2	FA3	FA4
(1) I am satisfied because I was able to learn what would be useful for my career as a pharmacist.	**0.87**	-0.11	-0.03	-0.05
(2) I was able to experience the overall work of a pharmacist sufficiently.	**0.75**	-0.10	0.01	0.05
(3) The pharmacists facilitated your appropriate self-learning so that you can solve the problems of the patients.	**0.71**	0.10	-0.02	0.03
(4) The pharmacists asked you related questions so that you can improve your ability to create an effective treatment plan.	**0.69**	0.09	0.00	0.01
(5) I felt that I had too many tasks that would not lead to learning for me as a trainee.	**-0.67**	0.03	0.05	0.03
(6) The pharmacists were able to have an empathetic communication with you.	**0.66**	-0.07	-0.04	0.14
(7) The pharmacists accepted you as a member of the team.	**0.64**	0.02	-0.07	0.07
(8) Some learning contents were far from pharmacist’s work.	**-0.62**	0.10	0.09	0.02
(9) The actual learning contents were in line with the learning objectives (SBOs).	**0.53**	-0.03	0.32	-0.03
(10) I was able to experience “Community-Based Care” at the community pharmacy sufficiently.	**0.46**	-0.01	0.05	0.08
(11) Safety precautions (medicine shelf arrangements, checking empty bags after mixed injection, etc.) were taken for medicine and injected drug preparation in general.	**0.45**	-0.04	0.13	-0.04
(12) The practical training contents were well matched with the pre-practice training.	**0.35**	0.30	-0.04	-0.13
(13) The university teachers gave you enough feedback on your learnings at the training site.	-0.15	**0.91**	0.01	0.06
(14) The university teachers provided enough support for me so that I can prevent trouble, or work on the practical training smoothly in case of trouble.	-0.06	**0.84**	0.02	0.04
(15) The practical training contents were well matched with the classes from the 1st to the 4th years (excluding the pre-practice training).	0.26	**0.38**	-0.04	-0.12
(16) Training schedule by day was prepared and informed to me in advance.	-0.03	0.01	**0.91**	-0.01
(17) Training schedule by hour was prepared and informed to me in advance.	0.00	0.03	**0.80**	0.01
(18) I was able to have enough time with patients to explain about drugs or disease.	0.13	0.03	-0.02	**0.83**
(19) I was able to have enough time with patients to talk with them.	0.06	0.07	0.02	**0.80**
Inter-factor correlations				
FA1		0.46	0.49	0.50
FA2			0.18	0.19
FA3				0.24
Cronbach's alpha	0.74	0.75	0.70	0.72

The factors were defined as the following: the first factor (FA1) was “satisfactory learning (pharmacy)”; the second factor (FA2) was the “support system of the university”; the third factor (FA3) was the “creation and clarification of the training plan”; and the fourth factor (FA4) was “dialogue with patients”

The first factor comprised 12 items including, “I am satisfied because I was able to learn what will be useful for my career as a pharmacist” and “I was able to experience the overall work of a pharmacist sufficiently.” This factor was interpreted to represent the satisfaction and sense of accomplishment of students regarding pharmacy practice programs, and was named “satisfactory learning (pharmacy).” The second factor comprised three items including, “The university teachers gave you enough feedback on your learnings at the training site” and “The university teachers provided enough support for me so that I can prevent trouble, or work on the practical training smoothly in case of trouble” and was interpreted to represent the educational content and student support provided by university teachers. This second factor was named “support system of the university.” The third factor comprised two items including, “Training schedule by day was prepared and informed to me in advance” and was interpreted to represent the creation of daily training plans and their clarification for students. This third factor was named “creation and clarification of the training plan.” The fourth factor comprised two items including “I was able to have enough time with patients to explain about drugs or disease” and was interpreted to represent the opportunities of getting involved with patients, and what transpired. This fourth factor was named “dialogue with patients.”

Next, the scores from all factors in 2011 and in 2012 were calculated. The following are the details of each score; “satisfactory learning” (mean ± SD in 2011: 4.278 ± 0.539, mean ± SD in 2012: 4.328 ± 0.571), “support system of the university” (mean ± SD in 2011: 3.727 ± 1.090, mean ± SD in 2012: 4.061 ± 1.005), “creation and clarification of the training plan” (mean ± SD in 2011: 3.843 ± 1.522, mean ± SD in 2012: 3.776 ± 1.516) and “dialogue with patients” (mean ± SD in 2011: 4.468 ± 1.194, mean ± SD in 2012: 4.461 ± 1.164), the average difference of all factors (mean ± SD in 2011: 16.32 ± 3.075, mean ± SD in 2012: 16.63 ± 2.928). We conducted a comparison of the scores derived from each factor for 2011 and 2012, for all four factors. The score was significantly higher for 2012 than 2011 for the item “support system of the university.” There were no significant differences found for the other factors or overall (the total of the each factor subscale score). The breakdown is listed as follows: “satisfactory learning (pharmacy)” (*t* = −1.630, *df* = 1075.799, *P* = 0.104, 95 % confidence interval [CI] = −0.110, 0.010); “support system of the university” (*t* = −5.655, *df* = 957.236, *p* < 0.001, 95 % CI = −0.450, −0.218); “creation and clarification of the training plan” (*t* = 0.783, *df* = 1022.322, *P* = 0.434, 95 % CI = −0.010, 0.232); “dialogue with patients” (*t* = 0.098, *df* = 1003.747, *P* = 0.922, 95 % CI = −0.123, 0.136); and overall (the total subscale of each factor) (*t* = −1.850, *df* = 983.734, *P* = 0.065, 95 % CI = −0.642, 0.019).

### Simple tabulation and chi-square test

We compared 2011 with 2012 by conducting simple tabulation and chi-square tests for 14 items, based on a two-point scale (Table 3). Results from the question presented on the review and discussion of opportunities among students at universities; a common question for hospital pharmacy and community pharmacy practice, had already been reported on, as part of the hospital pharmacy practice results and were therefore omitted. We found that the percentage of students who had the opportunity to discuss and review with each other at the training facility significantly increased from 27 % to 44 % (*p* < 0.01). Students that had the opportunity to visit other facilities comprised approximately 90 % of the total. It was noted that these opportunities assist students with achieving the learning objectives listed in the model core curricula of pharmacy practice programs, and to attain broad experience as a pharmacist. However, only 40 % of the students had the opportunity to discuss with other occupations (doctor, nurse, etc.) and 5 % of the students had the opportunity to socialize and discuss with students in other occupations. In addition, around 40 % of students responded that they had to repetitively perform simple, non-academic tasks, such as prescription sorting and inventory management of medicinal supplies. Moreover, it was found that disagreements and broken trust between students and patients, pharmacists, and other trainees had occurred, at ratios of around 2 %, 5 % and 1 %, respectively. There were also responses stating that around 7 % of students had received unreasonable treatment, such as being blamed for prescription errors and/or had been the target of angry outbursts by pharmacists.

**Table 3:** Results of the simple tabulation and the chi-square test

Question		2011	2012	*χ* ^2^(1)	*P*
(1) Was a patient assignment system involved in the drug administration guidance, which enabled you to take care of the same patients every time?	Yes	79(17)	153(18)	0.105	0.75, n.s.
	No	394(83)	718(82)		
(2) Did you have a time to have a discussion or a review among students at the training site?	Yes	133(27)	400(44)	41.597	<0.01
	No	365(73)	502(56)		
(3) Did you have opportunity to associate with other professionals (doctors, nurses, and others) to have discussions (including all the occasions such as conversation over the phone, communication with the medical team, or a casual daily talk in a ward)?	Yes	213(43)	371(41)	0.364	0.55, n.s.
	No	284(57)	533(59)		
(4) Did you have opportunity to associate with other students (medical students, nursing students, and others) to have discussions?	Yes	19(4)	44(5)	0.600	0.44, n.s.
	No	480(96)	862(95)		
(5) Did you have any opportunity to have a conference among pharmacists?	Yes	160(33)	250(28)	3.077	0.08, n.s.
	No	331(67)	645(72)		
(6) Did you have any opportunity to make a presentation at a conference among pharmacists?	Yes	63(13)	109(12)	0.081	0.78, n.s.
	No	430(87)	792(88)		
(7) Did you have any opportunity to visit other facilities?	Yes	455(91)	807(89)	0.874	0.35, n.s.
	No	44(9)	95(11)		
(8) Did you pay any training expense or training cost when you attended a practical work or training at the outside of the training site?	Yes	70(14)	95(11)	3.506	0.06, n.s.
	No	429(86)	809(89)		
(9) Did you have a time when you felt that you were forced to do simple tasks repeatedly?	Yes	192(39)	372(41)	0.674	0.41, n.s.
	No	303(61)	531(59)		
(10) Did you have any trouble with a patient?	Yes	6(1)	17(2)	0.530	0.47, n.s.
	No	493(99)	891(98)		
(11) Did you have any trouble with your pharmacist instructor?	Yes	27(5)	41(5)	0.356	0.55, n.s.
	No	472(95)	862(95)		
(12) Did you have any trouble among students?	Yes	2(0)	10(1)	1.136	0.29, n.s.
	No	497(100)	897(99)		
(13) Did you have any unreasonable occasions that you felt you got a tantrum from a pharmacist or were put the responsibility of a preparation error upon you during the training?	Yes	35(7)	53(6)	0.572	0.45, n.s.
	No	463(93)	853(94)		
(14) Did the community/ hospital pharmacy practice Program have an influence on your future career?	Yes	339(68)	590(66)	0.603	0.44, n.s.
	No	157(32)	302(34)		

Lastly, around 70 % of the students responded that community pharmacy practice has influenced their career choices; specifically with regards to the following items: “I believe the job of a pharmacist is appealing because working in a pharmacy allows us to get closer to patients, compared to in hospitals”; “I wanted to become a pharmacist like the instructor at the training facility”; “I want to work (or not work) at a pharmacy in future”; “I want to work at a pharmacy that also handles over-the-counter medical supplies rather than prescriptions only”; and “I want to become a pharmacist who can contribute to home health care/ community-based care.”

### McNemar’s test

We conducted McNemar’s test for 14 items, based on a two-point scale. Between 2011 and 2012, we found a significant increase in hospitals only for Pattern 1; a system that allowed the student to attend to the same patient for prescription instructions (hereafter, “charge system of patients”). However, there was a significant decrease in Pattern 4, in which students were unable to experience “charge system of patients” at neither hospitals nor pharmacies (*X*^2^ (3) = 14.33, *ps* < 0.01) (Table 4). Furthermore, there was a significant increase in Pattern 3, in which students had “opportunities to review and discuss with each other at training facilities” at both hospitals and pharmacies. Meanwhile, there was a significant decrease from 2011 to 2012 in students who did not get such opportunities at either hospitals or pharmacies (*X*^2^ (3) = 54.39, *ps* < 0.001). In addition, there was a significant decrease in Pattern 2, in which students at pharmacies only had the opportunity to visit other pharmacies, and a significant increase in Pattern 3, in which students had these opportunities at both hospitals and pharmacies (*X*^2^ (3) = 22.77, *ps* < 0.001). Moreover, there was a significant increase in Pattern 1, in which students were required to “take on the burden of paying the workshop participation fee, such as, to the Pharmacist Association” (*X*^2^ (3) = 20.3, *p* < 0.001), while there was a significant decrease in Pattern 2, in which students experienced the same, at pharmacies only (*X*^2^ (3) = 20.3, *p* < 0.05). Furthermore, there was no significant difference between the 10 other items.

**Table 4:** Results of McNemar’s test

Question		Pattern			
		1	2	3	4
		hosp. → Yes	hosp. → No	hosp. → Yes	hosp. → No
		comm. → No	comm. → Yes	comm. → Yes	comm. → No
(1) Was a patient assignment system involved in the drug administration guidance, which enabled you to take care of the same patients every time?	2011	▼170^**^	22	56	△220^**^
	2012	△388^**^	32	118	▼319^**^
(2) Did you have a time to have a discussion or a review among students at the training site?	2011	157	40	▼93^***^	△208^***^
	2012	263	84	△316^***^	▼238^***^
(7) Did you have any opportunity to visit other facilities?	2011	9	△327^***^	▼124^***^	35
	2012	22	▼478^***^	△327^***^	72
(8) Did you pay any training expense or training cost when you attended a practical work or training at the outside of the training site?	2011	▼11^***^	△53^*^	17	418
	2012	△67^***^	▼65^*^	30	742

The words were omitted as following: the hospital pharmacy practice is “hosp.”; the community pharmacy practice is “comm.”

*P < 0.05, **P < 0.01, ***P < 0.001

### Multiple choice question

The actual services of the pharmacist at each training site were clarified by the following multiple choice question: “Have you ever seen any of services of a pharmacist at the training site during training?” It was found that some students never saw how some pharmacist services, relating to the Model Core Curriculum for Pharmacy Practice were actually carried out. In particular, the rates of witnessing the services of pharmaceutical preparations, home health care, drug information service, public health and disaster medical care constituted only small portions, approximately 68 %, 64 %, 56 %, 54 % and 31 %, respectively.

### Free response

There was an overwhelming number of free responses stating that the training content and environment should be standardized so as to have no disparity for students or facilities, in terms of knowledge and experience. Additionally, a large number of responses indicated the need for a manual so that there would be no discrepancies between training content and any supplementary lessons conducted before and after training. The following opinions were also fairly frequent among students:

1. The continuity of clinical education and basic education

“Pre-practice training was only a formality, so I was perplexed on-site; the product names and side effects of medical supplies, transfusions, and interaction of injections should be learned beforehand.”

“Training was stimulating and was an important experience that I could not have had at university; I felt a strong sense of accomplishment as a pharmacist.”

“My motivation to become a pharmacist increased with on-site learning. My attitude towards studying changed.”

“I understood the difficulties of work on-site.”

2) The gap between the ideal and reality on the pharmacy practice program

“I wish the learning objectives matched the actual situation on-site more closely.” “I wanted to find out the training schedule beforehand.”

“The trainee receiving system was not ready.”

“At the hospitals, pharmacists were busy and trainees were left alone for long periods of time.”

“There was more classroom learning time at hospitals; thus, I was unable to participate in ward duties and team medical care as much.”

“In hospital pharmacy practice, particularly for bedside learning, doctors and nurses should collaborate beforehand so that training is more efficient and meaningful.”

“It was hard because pharmacists treated us like we were a nuisance.”

“Team medical care is only a formality and there is not much enthusiasm, with mostly conservative pharmacists.”

“For community pharmacy practice, we only got prescriptions from the clinics nearby/there were not many patients/because most of the duties were simple, I wasn’t able to learn much.”

3) The validity of the training period and learning content

“The community pharmacy practice period of two and a half months is too long.”

“Community pharmacy practice wastes too much time/because not much is actually learned, I requested that the learning content be enhanced, otherwise shorten the learning period should be shortened to one month, or have us rotate among multiple pharmacies.”

“For hospital pharmacy practice, because the training period is long, I was able to take care of a patient right from hospitalization to discharge; thus, I was able to learn the whole process. I am glad that I was able to continue to take care of a patient for a whole month.”

4) The ingenuity of timing and opportunity for pharmacy practice program

“I wish I could have experienced training earlier in my studies at school.”

“I want them to listen to my requests regarding at which facility the training should be conducted.”

“I want to build experience by training at more hospitals and pharmacies.”

“For the joint training (in certain regions, trainees gathered together and learnt content regarding over-the-counter sales, preparations manufactured by pharmacies, preparation of Chinese medicine, and the activities of school pharmacists, etc.) during the community pharmacy practice, I was able to get along well and share information with students from other universities. The joint training in the city provided a good incentive.”

5) Others

“It was a good opportunity to consider my vision for the future.”

“I wish they had treated me as a trainee and not a part-time worker.”

## Discussion

For the traditional four-year pharmaceutical education program, pharmacy practice programs were either prerequisites or could be taken as electives. The training period varied between universities [3]. Furthermore, the training content varied widely between training facilities. Because of this, a training plan was created with a model core curriculum for pharmacy practice programs, in order to ensure the quality and homogeneity of the programs conducted at each university. The evaluation of model core curriculum for pharmacy practice programs and pharmaceutical education began in 2011. A “revised model core curriculum for pharmaceutical education (hereafter, revised model core curriculum)” was created [22]. Moreover, the revised model core curriculum will be implemented for freshman from 2015. In 2019, when these freshmen will be in their 5^th^ year, we are scheduled to conduct pharmacy practice programs that utilize the revised model core curriculum. However, it is still important to understand and improve on the current pharmacy practice programs conducted nationwide in Japan. This will help to enhance the educational effect of the current programs that are scheduled to continue until 2018. This will be in addition to the pharmacy practice programs that will use the revised model core curriculum scheduled to begin in 2019. Due to the above, we aimed to understand the actual state of nationwide pharmacy practice programs.

In the previous study [21], we evaluated hospital pharmacy practice. In this community pharmacy practice, we used the same questionnaire as the evaluation of hospital pharmacy practice, because various stakeholders and parameters that are thought to be related to the educational effects of the whole pharmacy practice programs were incorporated.

Initially, it was expected that there would be discrepancies in the training content between hospitals and community pharmacies due to the range of their work differs. As such, we extracted the performance index for each type of training based on exploratory factor analysis. The following four factors were extracted for community pharmacy practice: “satisfactory learning (pharmacy),” “support system of the university,” “creation and clarification of the training plan,” and “dialogue with patients.” Note that regardless of the same questionnaire being used for hospital pharmacy practice and community pharmacy practice, in the end, a different factor was extracted from each practice. “Creation and clarification of the training plan,” which comprised item such as creating a schedule on a daily/hourly basis and clarifying the schedule to students was extracted for community pharmacy practice. In contrast, “support system of the training site (hospital)” comprising item such as communication and relationships with pharmacists was extracted for hospital pharmacy practice. Each of factors indicated a moderate positive correlation for “satisfactory learning (hospital/pharmacy).” The correlation between these factors was 0.67 and 0.49 for learning at hospital pharmacies and community pharmacies, respectively.

From the above, the qualitative difference in training content experienced in hospital pharmacy practice and community pharmacy practice was presumed to have influenced the evaluations made by the students. In other words, As the reason of extracted “support system of the training site (hospital),” this is thought to have resulted from the state of hospital pharmacy practices, as judged by the coordination and support of pharmacists, which requires students to participate in various learning locations, including clinical conferences and team medical care (palliative care team and infection prevention and control team, etc.). Moreover, as to the reason for the extracted “creation and clarification of the training plan,” there are many cases where community pharmacy practices accept students at pharmacies that specialize in handling pharmacy services under health insurance. It is possible to consider the work at these pharmacies involves simple and repetitive tasks, and it is presumed that the training content of these practices may be poor.

In other words, for hospital pharmacy practice, pharmacists should review communication and treat students in a respectful manner, as it relates to “support system of the training site (hospital).” It is also important to confirm whether there are any differences in the instruction content and quality for students because of the difference in student compatibility and level of communication. Moreover, the free responses (“I was left alone,” “I was treated as a nuisance,” etc.) seem to indicate a hostile training environment. It is therefore inferred that we need to reexamine the system to ensure students are received with a warm welcome. For community pharmacy practice, pharmacists should confirm a certain training schedule is created to ensure that students experience all pharmacy duties, and explained to the students in relation to “creation and clarification of the training plan.” Furthermore, when reviewing the free responses of the students (“the period of two and a half months is too long,” “not much content is actually learned,” etc.), the developers of the model curriculum for pharmacy practice programs are required to evaluate in detail whether the training period and learning objectives of the curricula appropriately correspond to the actual situations at the medical sites (especially pharmacies). This would, in all likelihood, result in significant improvements being made to the content of the pharmacy practice programs.

In Pharmacist Training Workshop for Pharmacy Practice Instructor, it may also be effective to enforce content relating to interaction with students and the appropriate preparation of training schedules. Furthermore, at present, one instructor pharmacist has completed the workshop is assigned at each training facility. However, in reality, it is impossible for one instructor pharmacist to instruct all students. Therefore, increasing the number of assigned instructor pharmacists and recommending the certification of instructor pharmacist to all pharmacists could lead to improvements in the current state of pharmaceutical training.

In addition, for community pharmacy practice, we found that there was no change in the mean values of each factor extracted by factor analysis between 2011 and 2012, with the exception of the “support system of the university.” Furthermore, there was no change over the 2 years, even for the total of each factor subscale score. In other words, no change was found in the content or quality of community pharmacy practice.

From the results of simple tabulation and the chi-square test, the following was found for community pharmacy practice: for both years, around 90 % of students stated they had had an opportunity to visit other facilities to accumulate broader experience as a pharmacist and accomplish all learning objectives listed in the model core curriculum for the pharmacy practice programs (in 2011: 91 %, in 2012: 89 %). At the introduction of the pharmacy practice programs, it was a requirement to form a group with multiple facilities, accept students, and standardize training content. However, this would have necessitated tremendous effort for pharmacists in community pharmacy practice. Furthermore, in the free responses, students from certain regions frequently mentioned the joint training and evaluated it as useful and effective. On the other hand, due to the many requests to standardize the training content nationwide, the joint training may be effective for correcting disparities in the teaching system and learning content at each community pharmacy. However, many students have requested standardization of training content and environment for both hospital pharmacy practice and community pharmacy practice. This could be due, in part, to the fact that evaluation of accomplishing such standardization for learning objectives is ambiguous. Thus, it may be more effective to introduce fill-in-the-blank style training books which are used in countries such as England, and to have training passes evaluated by Kubo et al [20].

Furthermore, certain points that have reinforced the moderate positive correlation found between “satisfactory learning” and “dialogue with patients” were found in both hospital pharmacy practice and community pharmacy practice (correlation between the factors was 0.53 and 0.50, respectively) [21]. Before introducing pharmacy practice programs, the importance of participatory training rather than observational learning was mentioned, with the objective of creating a connection between students and their patients. Furthermore, university instructors participating in the development of the model core curriculum for pharmacy practice programs have engaged in educational activities targeting all university instructors and pharmacists at training facilities. Therefore, the pharmacy practice programs are now more closely matched to the students’ needs. It was indicated that promoting such programs leads to “satisfactory learning,” accompanied by student satisfaction and a sense of accomplishment and should therefore be continued in future.

From the results of McNemar’s test, from 2011 to 2012, there was a significant increase in the number of students who were able to experience the “charge system of patients” at hospitals only, and a significant decrease among students who were unable to experience this system, at neither hospitals nor pharmacies (*ps* < 0.01).

The results of factor analysis (correlation between “satisfactory learning” and “dialogue with patients” was 0.53 in hospital pharmacy practice, and 0.50 in community pharmacy practice) and the above results of McNemar’s test suggest that opportunities are being created for interaction with patients. As such, the effectiveness of assigning a patient to each trainee is reinforced, along with in-depth pharmaceutical care. This is in addition to enhancing professionalism and responsibility as a pharmacist. When reviewing the free responses of the students (“I am glad that I was able to continue to take care of a patient for a whole month”) and the above results of McNemar’s test, it is also effective to establish a certain training period (especially in hospitals) and conduct training at multiple facilities. Furthermore, it was indicated that it would be effective to retain training at both hospitals and pharmacies and continue to make improvements to both training content and readiness to receive trainees on a daily basis. This would help to correct and mutually supplement disparities such as those between training facilities and educational content.

The revision of the model core curriculum for pharmaceutical education and pharmacy practice programs has led to these becoming unified. Additionally, reinforcing the continuity of clinical education centered on medical settings and basic education centered on the university has been promoted. However, it was inferred from reviewing the free responses (“team medical care was only a formality,” “there is not much to learn at the pharmacy,” etc.) that students in the current pharmacy practice programs are learning something of an unintended or negative “hidden curriculum” based on the gap between the ideal and the reality of the experience. To reduce these factors and to train pharmacists with basic knowledge, skills, and positive attitudes for contributing to team medical care and community-based care, it appears that cooperative student training is required. To accomplish this, stakeholders within pharmacy practice programs in hospitals, pharmacies, and universities must communicate with each other in order to deepen their mutual understanding of the training required.

## Conclusion

The following four factors were extracted for community pharmacy practice: “satisfactory learning (pharmacy),” “support system of the university,” “creation and clarification of the training plan,” and “dialogue with patients.” From 2011 to 2012, there were no significant differences found in the factors, with the exception of the “support system of the university.” Moreover, to accomplish the learning objectives, the community pharmacy practice program has introduced some initiatives, such as joint training. Furthermore, conducting training at multiple facilities deepens student learning and assists with the correction of problems, such as the disparities within the teaching system and learning content at each of the training facilities.
